# Equipping Durum Wheat—*Thinopyrum ponticum* Recombinant Lines With a *Thinopyrum elongatum* Major QTL for Resistance to Fusarium Diseases Through a Cytogenetic Strategy

**DOI:** 10.3389/fpls.2019.01324

**Published:** 2019-10-22

**Authors:** Ljiljana Kuzmanović, Giulia Mandalà, Silvio Tundo, Roberto Ciorba, Matteo Frangella, Roberto Ruggeri, Francesco Rossini, Federica Gevi, Sara Rinalducci, Carla Ceoloni

**Affiliations:** ^1^Department of Agricultural and Forest Sciences (DAFNE), University of Tuscia, Viterbo, Italy; ^2^Department of Ecological and Biological Sciences (DEB), University of Tuscia, Viterbo, Italy

**Keywords:** alien gene transfer, chromosome engineering, chromosome pairing, GISH, marker-assisted selection, Triticum, wild wheat relatives, sustainability

## Abstract

Prompted by recent changes in climate trends, cropping areas, and management practices, *Fusarium* head blight (FHB), a threatening disease of cereals worldwide, is also spreading in unusual environments, where bread wheat (BW) and durum wheat (DW) are largely cultivated. The scarcity of efficient resistance sources within adapted germplasm is particularly alarming for DW, mainly utilized for human consumption, which is therefore at high risk of kernel contamination by health-dangerous mycotoxins (e.g., deoxynivalenol = DON). To cope with this scenario, we looked outside the wheat primary gene pool and recently transferred an exceptionally effective FHB resistance QTL (*Fhb-7EL*) from *Thinopyrum elongatum* 7EL chromosome arm onto a *Thinopyrum ponticum* 7el_1_L arm segment, containing additional valuable genes (including *Lr19* for leaf rust resistance and *Yp* for yellow pigment content), distally inserted onto 7DL of BW lines. Two such lines were crossed with two previously developed DW-*Th. ponticum* recombinants, having 7el_1_L distal portions on 7AL arms. Genomic *in situ* hybridization (GISH) analysis showed homologous pairing, which is enabled by 7el_1_L segments common to the BW and DW recombinant chromosomes, to occur with 42-78% frequency, depending on the shared 7el_1_L amount. Aided by 7EL/7el_1_L-linked markers, 7EL+7el_1_L tetraploid recombinant types were isolated in BC_1_ progenies to DW of all cross combinations. Homozygous 7EL+7el_1_L recombinant plants and null segregates selected in BC_2_F_2_ progenies were challenged by *Fusarium graminearum* spike inoculation to verify the *Fhb-7EL* efficacy in DW. Infection outcomes confirmed previous observations in BW, with >90% reduction of disease severity associated with *Fhb-7EL* presence *vs*. its absence. The same differential effect was detected on seed set and weight of inoculated spikes, with genotypes lacking *Fhb-7EL* having ∼80% reduction compared with unaffected values of *Fhb-7EL* carriers. In parallel, DON content in flour extracts of resistant recombinants averaged 0.67 ppm, a value >800 times lower than that of susceptible controls. Furthermore, as observed in BW, the same *Fhb-7EL* also provided the novel DW recombinants with resistance to *Fusarium* crown rot (∼60% symptom reduction) as from seedling infection with *Fusarium culmorum*. Through alien segment stacking, we succeeded in equipping DW with a very effective barrier against different *Fusarium* diseases and other positive attributes for crop security and safety.

## Introduction

With about 8% coverage of the world’s wheat area, durum wheat (*Triticum durum* Desf., 2n = 4x = 28, genome AABB) is the 10th most important crop in the world (Bassi and Sanchez-Garcia, 2017). Not only does it represent a strategic commodity for the three world areas where it is mainly cropped (the Mediterranean basin, the North America’s Great Plains, and the desert areas of South-Western United States and Northern Mexico; Ranieri, 2015), but durum wheat cultivation is also expanding in Canada, India, and even the Senegal River basin in sub-Saharan Africa (Sall et al., 2018). As with all other crops, it is experiencing the effects of climate changes, hence requiring dedicated breeding efforts to cope with them and concurrent challenges to the present and projected demand for higher food supply (e.g., Ray et al., 2019).

As a result of climate extremes, particularly rising temperatures, not only do the conventional distribution areas of crops tend to be modified (e.g., Ceoloni et al., 2014a), but the ecology, epidemiology, and virulence/aggressiveness of their pathogens are also subject to considerable variation (Fones and Gurr, 2017). Plants suffering abiotic stresses such as heat and drought are more susceptible to unspecialized necrotrophic pathogens, the same stress conditions also accelerating pathogen evolution (Chakraborty, 2013; Vaughan et al., 2016). Typical necrotrophs are fungal pathogens belonging to the *Fusarium* genus, responsible for some of the most threatening diseases of wheat and other cereals, namely, Fusarium head blight (FHB) and Fusarium crown rot (FCR). Environments where humid and warm conditions occur around the flowering stage are typically prone to FHB, while FCR is prevalent under drier conditions. On a world scale, FHB is predominantly caused by *F. graminearum*, while *F. culmorum* and *F. pseudograminearum* are the main agents of FCR (Gilbert and Haber, 2013; Scherm et al., 2013; Matny, 2015). They are all toxigenic fungi, secreting secondary metabolites that play a significant role in pathogen virulence *in planta*, likely due to their ability to inhibit eukaryotic protein synthesis (reviewed in Bakker et al., 2018). In wheat, the most frequently detected of such mycotoxins is deoxynivalenol (DON), belonging to the trichothecenes, whose role as virulence factor in FHB and FCR was consistently demonstrated in bread and durum wheat subjected to inoculation with the *Fusarium* species mentioned earlier (Mudge et al., 2006; Scherm et al., 2011; Sella et al., 2014; Mandalà et al., 2019). Alongside its role in pathogenesis, DON is a highly hazardous compound for human and animal health (Maresca, 2013), and strict rules and legislative limits for maximum levels in food and feed have been defined worldwide (Romer Labs Division Holding GmbH, 2016). The economic value of contaminated crops is affected not only by safety problems but also by grain yield and quality penalties, due to failed development or shrivelling, discoloration, and low test weight of infected kernels (e.g., McMullen et al., 2012; Matny, 2015; Salgado et al., 2015).

Impacts on safety, security, and processing issues are particularly alarming for durum wheat, used almost exclusively for transformation into human food products. In a sustainable agricultural perspective, and also considering that agronomic practices and fungicides can only partially reduce the infection risks, the use of resistant cultivars is widely recognized as the most effective tool for controlling Fusarium diseases (e.g., Steiner et al., 2017). However, the needed genetic variation for successful breeding actions addressing such diseases appears to be quite scarce within the cultivated and closely related tetraploid gene pools, being limited to quantitative trait loci (QTL) of minor individual effect (Prat et al., 2014).

In bread wheat (*Triticum aestivum* L., 2n = 6x = 42, genome AABBDD), breeding for FHB resistance has so far been centered mostly on a large-effect QTL, namely *Fhb1*, located on the 3BS chromosome arm of the bread wheat Chinese cultivar Sumai 3 and its derivatives (Gilbert and Haber, 2013; Steiner et al., 2017). Similarly, a single major QTL, identified on 3BL of hexaploid germplasm (*T. spelta*), is being exploited for FCR resistance breeding (Liu and Ogbonnaya, 2015). Being located on a shared chromosome, i.e., 3B, transfer of both QTL from bread wheat into durum wheat represented a relatively amenable option. However, results indicated dependency on the cultivar background for the expression of *Fhb1*-linked resistance (Prat et al., 2017), with lack of any FCR improvement associated with presence of the 3BL locus (Ma et al., 2012). Whether the higher susceptibility of durum wheat than bread wheat toward Fusarium diseases, and hence the partial and unpredictable effect of interspecific transfers, might be due to durum wheat-specific susceptibility factors, or to the so far minor exposure of the crop to relevant disease pressure, remains to be elucidated (Giancaspro et al., 2016). No doubt, the current lack of highly resistant genotypes among cultivated durum wheat worldwide is also the result of limited breeding efforts to date targeting *Fusarium* spp. resistance in durum wheat compared with bread wheat (Giancaspro et al., 2016; Prat et al., 2017).

A wide array of beneficial traits, rarely or not represented in cultivated wheat or closely allied gene pools, such as resistance to Fusarium diseases, resides in more distant relatives, including perennial Triticeae of the *Thinopyrum* genus (Forte et al., 2014; Ceoloni et al., 2015 and references therein). Belonging to the wheat tertiary gene pool, they still retain considerable cytogenetic affinity with wheat chromosomes, albeit often characterized by segmental homoeology (Ceoloni et al., 2015). A meaningful example of positive impact of a *Thinopyrum* source on enhancement of *Fusarium* spp. resistance in cultivated wheat germplasm is that of a major QTL, named *Fhb-7el* *_2_* (Forte et al., 2014) and later *Fhb7* (Guo et al., 2015b), originating from chromosome 7el_2_ of decaploid *Th. ponticum*. This strong FHB resistance QTL was pyramided into bread wheat (Shen and Ohm, 2007; Zhang et al., 2011; Forte et al., 2014) and also durum wheat (Forte et al., 2014) by exploiting the close relatedness of 7el_2_ with 7el_1_ (Dvorak, 1975; Guo et al., 2015a), deriving from a different accession of the same species. The latter bear genes for effective rust resistances (*Lr19* and *Sr25*) and for yield-contributing traits (see Ceoloni et al., 2015 for a review). In both wheat species, the effect of the 7el_2_ QTL was highly significant when compared with susceptible sibs, reducing FHB severity on infected spikes by 70–85% (Forte et al., 2014).

Previously obtained wheat-alien translocation and recombinant lines with portions of the respective *Thinopyrum* donor chromosome containing the target genes were instrumental to the successful pyramiding of the 7el_2_+7el_1_ genes/QTL *via* 7el_1_–7el_2_ pairing and recombination. These wheat-alien primary transfer lines were, in turn, the result of chromosome engineering, i.e., a suite of cytogenetic methodologies which enable alien segmental introgressions into wheat homoeologous chromosomes, mostly *via* pairing mediated by wheat *ph1* mutations (reviewed in Ceoloni and Jauhar, 2006; Qi et al., 2007). However, in wheat-alien combinations, *ph1* mutations promote autosyndetic (wheat–wheat), as well as allosyndetic (wheat–alien) homoeologous chromosome pairing and recombination. This represents a drawback, which limits recovery and affects background stability of target allosyndetic recombinants (see, e.g., Ceoloni and Jauhar, 2006; Zhang et al., 2017).

**Figure 1:**
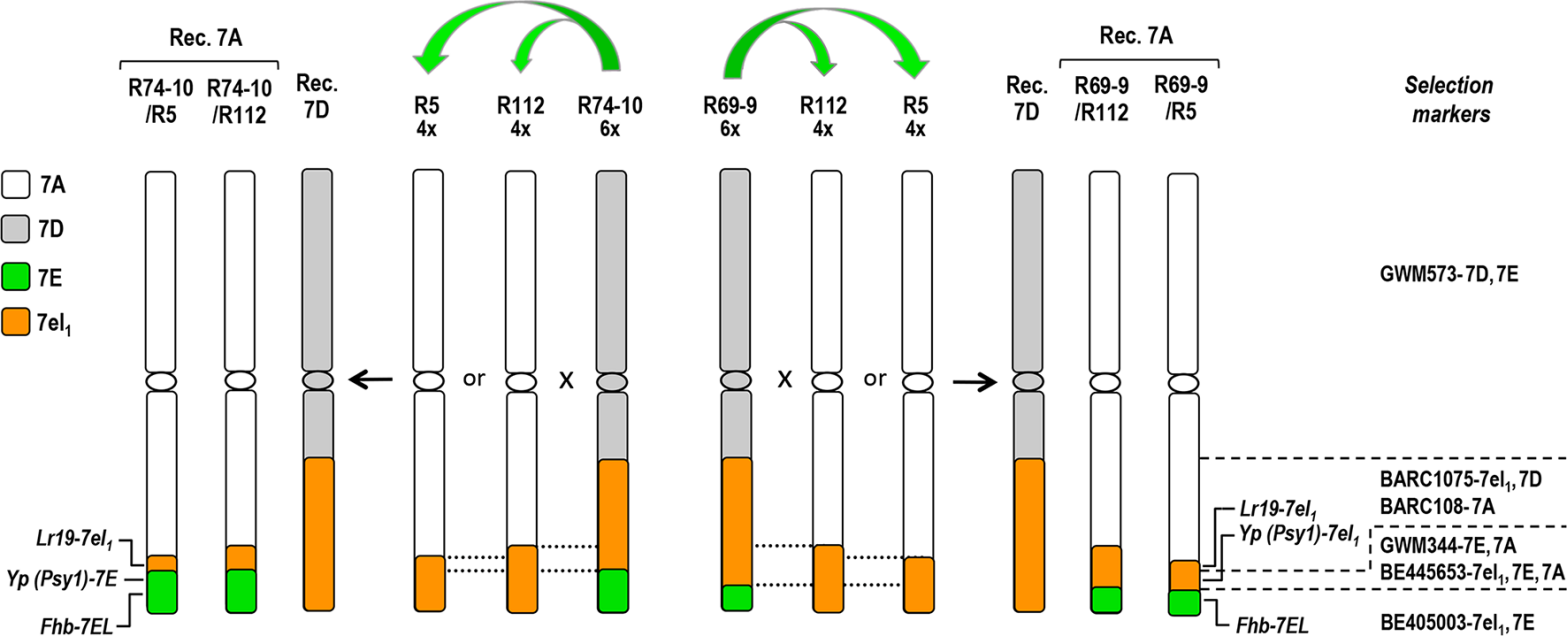
Cytogenetic maps of group-7 wheat-*Thinopyrum* spp. chromosomes involved in the pyramiding scheme of distal 7EL segments carrying the *Fhb-7EL* QTL (from 6x lines R69-9 or R74-10) into 7el_1_L-7AL arms of 4x lines R112 or R5. Dotted lines delimit the regions where homologous pairing and crossing-over in the shared 7el_1_L regions can occur and give rise to the desired pyramiding. Dashed lines on the right indicate the chromosomal regions where marker loci and target genes are located. For detailed genetic and physical mapping data of *Thinopyrum* spp. segments into wheat chromosomes, see Ceoloni et al., 2017a.

To circumvent these problems, an alternative strategy was followed in the recent transfer of a major QTL for resistance to Fusarium diseases from chromosome 7E of *diploid Th. elongatum* into bread wheat (Ceoloni et al., 2017a). This did not rely on the *ph1* promotion but took advantage of the close homoeology relating *Th. elongatum* chromosome 7E and *Th. ponticum* 7el_1_ (Dvorak, 1975). As a result, spontaneous pairing and recombination occurred between the 7E long arm (7EL) and a sizable 7el_1_L segment (70% of the arm length), present in a 7E(7D) substitution line and in the 7DS·7DL-7el_1_L of the T4 translocation line, respectively. Pyramiding of the positive traits controlled by 7el_1_L genes/QTL (see above) with the 7EL-linked Fusarium resistance QTL (named *Fhb-7EL*) was thus achieved, with *Fhb-7EL* being shown to map more distally than the 7el_1_L genes (Ceoloni et al., 2017a). The presence of small 7EL terminal segments containing the *Fhb-7EL* QTL was shown to determine an exceptionally effective FHB resistance in bread wheat recombinant lines inoculated with *F. graminearum*, of the same degree as that previously associated with the entire 7E or 7EL (Shen et al., 2004; Miller et al., 2011). The “type II” resistance, i.e., resistance to fungal spread within host tissues (Mesterházy et al., 1999), was expressed at its maximum level, with spread of the pathogen limited to the immediate vicinity of the inoculated floret (Ceoloni et al., 2017a), and an average 95% reduction of disease severity in inoculated spikes of *Fhb-7EL* carrier *vs*. non-carrier lines. In the same work, the *Fhb-7EL* QTL was for the first time also demonstrated to substantially reduce FCR, incited by seedling inoculation with *F. culmorum* and *F. pseudograminearum*. Marker- and phenotype-based assessments showed some of the recombinants bearing *Fhb-7EL* to possess additional desirable 7el_1_L genes. In particular, *Fusarium* spp. resistant recombinant lines R69-9 and R74-10 were shown to include in their proximal 7el_1_L segments the leaf rust resistance gene *Lr19* and different alleles at the *Psy1* (*Phytoene synthase 1*) locus (*Psy1-7el* *_1_* *L* in R69-9, and *Psy1-7EL* in R74-10; see **Figure 1**), consistently associated with increases of yellow pigment (*Yp*) content (Zhang and Dubcosky, 2008; Ceoloni et al., 2017a).

For their chromosomal and genetic makeup, as well as the good agronomic performance in preliminary tests (Ceoloni et al., 2017a), bread wheat recombinant lines such as R69-9 and R74-10 appeared as attractive candidates for the incorporation of the 7EL+7el_1_L gene/QTL package into durum wheat as well. The envisaged strategy was to rely on potential homologous pairing of such donor chromosomes with recipient ones sharing some 7el_1_L chromatin. These were present in previously obtained 7AL-7el_1_L durum wheat introgression lines (Ceoloni et al., 2005). Among them, lines R5 and R112 not only possess the *Lr19*+*Sr25*+*Yp* genes from 7el_1_L but also showed 7el_1_L-linked positive effects on various yield components in various environments (Kuzmanović et al., 2014, Kuzmanović et al., 2016, Kuzmanović et al., 2018). The 7el_1_L segment spans 23% of the recombinant 7AL in R5 and 28% in R112 (Ceoloni et al., 2005). In principle, the same scheme adopted for the bread wheat transfer, i.e., use of the bread wheat 7E(7D) substitution line as donor of the *Fhb-7EL* resistance QTL, might have been attempted for its introduction into 7el_1_L segments of R5 and R112. However, previous experience suggested this route to be quite impractical for durum wheat. In fact, mainly due to the different chromosomal contexts (pentaploid *vs*. hexaploid hybrids), spontaneous pairing between the 7EL arm from the 7E(7D) substitution line and the 7el_1_L segments of R5 or R112 was expected to be sharply reduced compared with that using the T4 translocation line, as observed in the aforementioned 7el_1_L+7el_2_L pyramiding (Forte et al., 2014).

The objectives of the work described here were (i) to engineer the R5 and R112 7el_1_L segments with telomeric 7EL portions, bearing the *Fhb-7EL* QTL, by exploiting the homologous pairing potential of 7el_1_L segments shared by recipient and donor chromosomes; (ii) to verify the ability and extent of the *Fhb-7EL* QTL in conferring FHB and FCR resistances once stably introgressed into the new genomic context of durum wheat; and (iii) to provide a preliminary assessment of stability and performance of novel recombinant types, in relation to their exploitation in breeding.

## Materials and Methods

### Plant Materials and Transfer Scheme

Donors used for the transfer of the *Th. elongatum Fhb-7EL* QTL into durum wheat were two bread wheat recombinant lines, named R74-10 and R69-9 (7DS·7DL-7el_1_L/7EL; see **Figure 1**), with a terminal 7EL portion, including *Fhb-7EL*, embedded into a 7el_1_L *Th. ponticum* segment extending to 0.7 fractional length of the 7DL arm (T4 translocation line; see Ceoloni et al., 2017a). To combine the *Fhb-7EL* locus with 7el_1_L-linked positive genes/QTL for durum wheat performance (see, e.g., Gennaro et al., 2003, Gennaro et al., 2007; Kuzmanović et al., 2014, Kuzmanović et al., 2016), R74-10 and R69-9 were each crossed with two *Th. ponticum*–durum wheat recombinant lines, named R5 and R112, having 23 and 28%, respectively, of their distal 7AL arms replaced by homoeologous 7el_1_L portions (7AS·7AL-7el_1_L; see **Figure 1**) in a background near-isogenic to that of the Italian durum wheat cv. Simeto (Ceoloni et al., 2005). Pentaploid (5x) hybrid progeny of each of the four cross combinations was then subjected to further cross with cv. Simeto, hence consisting of backcrosses (BCs) to the same recurrent background. To identify recombinant types, BC_1_ plants (e.g., R74-10/R112//Simeto = R74-10/2*T. durum) were analyzed by suitable polymorphic markers (**Figure 1**), and the chromosome number of recombinant individuals determined (see below). BC_2_ progenies were then obtained from plants whose marker profile was indicative of the location on 7AL of the targeted 7EL+7el_1_L assembly, and these, with the majority having reached a euploid condition (2n = 28), were self-pollinated. BC_2_F_2_ offsprings were then genotyped, and the resulting homozygous carriers (HOM+) and non-carriers (HOM–) of the specific 7EL+7el_1_L combination, as well as their self-fertilized progeny, were used in various comparative tests. In these, depending on the type of experiment, the Chinese Spring (CS) 7E(7D) substitution line (2n = 42), original donor to R74-10 and R69-9 of the *Fhb-7EL* QTL (see Ceoloni et al., 2017a), the R112 and R5 recombinants, as well as durum wheat cv. Simeto, were included as control lines. Data from R112 and R5 plants (FHB infection and subsequent assays on inoculated plants, see below) were pooled (hereafter indicated as R112+R5), as the two genotypes did not show appreciable differences for such traits (see, e.g., Forte et al., 2014).

### Cytogenetic Analyses

Standard Feulgen or aceto-carmine staining techniques were applied both to assess the somatic chromosome number in root tip cells of selected genotypes and for quick anther screening from freshly collected young spikes, in view of meiotic metaphase I preparations. To this aim, selected anthers with pollen mother cells (PMCs) at the target phase, kept at −20°C in 3:1 fixative (absolute alcohol:acetic acid) for up to several weeks, were rinsed in 45% acetic acid, transferred for about 1 h to 2% aceto-carmine in 60% acetic acid at 37°C and squashed in 45% acetic acid, before freezing the slide in liquid nitrogen. For pairing analyses, metaphase I spreads were subjected to GISH (genomic *in situ* hybridization), using total DNAs of *T. aestivum* and *Th. ponticum* as genomic probes. Due to the close relatedness between *Th. elongatum* and *Th. ponticum* genomes, the latter equally highlights any *Thinopyrum* spp. introgression into wheat. Total DNAs were extracted from leaves following Tai and Tanksley (1990), mechanically sheared to 8–10 kb fragments and labeled by nick translation, including biotin-11-dUTP (Fermentas) or digoxigenin 11-dUTP (Roche Diagnostics) in the deoxyribonucleotide (dNTP) mix. The hybridization protocol followed Anamthawat-Jónsson and Reader (1995) with some modifications. In particular, to enrich the hybridization mixture in genome-specific sequences, equal quantities (100 ng) of denatured and differently labeled wheat and *Thinopyrum* probes were allowed to preanneal for 30 min at 58°C. Prior to hybridization with the pre-annealed probes, a blocking mixture, containing 1 mg of autoclaved and unlabeled DNA of *Aegilops speltoides* (2n = 14, genome SS, closely related to the B genome of polyploid wheats), was applied for 1.5 h at 63°C onto denatured chromosome preparations. This additional step led to a preferential block of B-genome chromosomes (not involved in wheat-*Thinopyrum* rearrangements), which enhanced the overall differentiation among chromosomes/segments of different genomic origin. Hybridization was then carried out for 2 h at 63°C, after which digoxigenin- and biotin-labeled probes were correspondingly detected using anti-digoxigenin conjugated with FITC (Roche; green fluorescence) and streptavidin conjugated with Cy3 (Amersham; red fluorescence).

All chromosome preparations were analyzed using a Leica DM5000B epifluorescence microscope, equipped with a SPOT-RT3 (Diagnostic Instruments, Inc.) color digital camera and the SPOT™ Advanced Plus imaging software.

### Marker-Assisted Selection (MAS)

The choice of suitable markers enabling discrimination between parental and recombinant types in BC_1_ progeny (pentaploid F_1_s × Simeto) and further genotyping in subsequent generations was facilitated by previously established inter-genomic polymorphism and genetic/physical mapping of several wheat and *Thinopyrum* spp. group 7 markers in the chromosomal regions of interest (see Ceoloni et al., 2017a, Ceoloni et al., 2017b and additional references therein). Therefore, only a limited number of PCR-based, mostly codominant markers were employed (**Figure 1**, **Table 1**). Most of these markers were used to isolate and confirm identity of recombinant types in the BC_1_ progeny to Simeto, while only one (e.g., BE405003) was sufficient to select for heterozygous recombinants in all BC_2_ progenies (presence of the 7E allele), and to discriminate heterozygotes (HET) from HOM+ and HOM− for the 7EL+7el_1_L segment assembly in BC_2_F_2_ progenies of 7A recombinants (e.g., BE445653 or GWM344). The STS*Lr19*_130_ marker, closely linked to *Lr19* (Prins et al., 2001), was employed to confirm presence of the *Th. ponticum* leaf rust resistance gene. A previously developed STS-CAPS assay (Ceoloni et al., 2017a), enabling discrimination of 7EL *vs*. 7el_1_L alleles, was applied to tag the *Psy1* gene, associated to the *Yp* phenotype (see Introduction).

**Table 1:** Group 7 molecular markers used to identify wheat—*Thinopyrum* spp. genotypes in the course of the work.

Marker	Type	Primer concentration (nM)	Other reagents	Annealing temperature (°C)	Alleles amplified (bp)					
					7el_1_L	7ES	7EL	7AL	7DS	7DL
BE405003	EST	400	–	55	700	–	600	–	–	–
BE445653	EST	250	5% DMSO	52	930	–	1200	750	–	–
GWM344	SSR	200	–	55	–	–	100	130–150	–	–
GWM573	SSR	200	–	50	–	200	–	–	180	–
BARC1075	SSR	200	– –	53 53	250	–	–	–	–	200
BARC108	SSR	250			–	–	–	160	–	–
STS*Lr19*_130_	STS	200	–	58	130	–	–	–	–	–
STSPsy1	STS-CAPS	200	–	60	730	–	705	450+270	–	–

BARC1075 and BARC108 markers were used in a multiplex assay (see Materials and Methods); for details of the CAPS assay applied for STSPsy1, see Ceoloni et al., 2017a.

For PCR reactions, DNA was extracted from young leaves or half-kernels according to Dellaporta et al. (1983). Primer sequences were retrieved from the public GrainGenes databases (http://wheat.pw.usda.gov/GG3/). For each 10 μl PCR reaction, 1× GoTaq^®^ G2 Master Mix (Promega, #M7822) and 25 ng of DNA were used for all primer pairs, while primer concentration, annealing temperature, and use of additional reagents varied, as reported in **Table 1**. Except for BARC1075 and BARC108 markers, for which a multiplex assay was developed, all other markers were amplified in a simple PCR. Amplified products were separated on 1.5–3% agarose gel, visualized by ethidium bromide staining and images captured with Kodak EDAS 290 digital system.

### *Fusarium* spp. Inoculation and DON Assays

#### FHB: Spike Inoculations With *F. graminearum*

Tetraploid homozygous carriers (HOM+) and non-carriers (HOM−) of the *Fhb-7EL* locus (based on marker analyses), isolated in BC_2_F_2_ progenies after crossing with Simeto (see above) of R74-10/R112, R74-10/R5 and R69-9/R112 F_1_s, together with the recurrent parent cv. Simeto, as well as R112 and R5 recipient lines and the CS7E(7D) substitution line (as FHB resistant control), were employed for a single-floret *F. graminearum* inoculation experiment. The R69-9/R5 corresponding progeny was not available at the time of inoculations, hence was not included in the assay. The infection assay was conducted under controlled conditions (16 h light/8 h dark photoperiod and 22–24°C/20°C corresponding temperature regimes) when plants were at mid-anthesis stage. The inoculum consisted of 1,000 macroconidia of *F. graminearum* strain 3824 (Tundo et al., 2016), freshly cultured on synthetic nutrient agar (SNA) medium (Urban et al., 2002), suspended in 20 μl of sterile distilled water (5 × 10^4^ ml^−1^ concentration), and supplemented with 0.05% Tween-20. The conidia suspension was pipetted through the glumes onto the basal floret of one central spikelet from the tip of the first spike of each plant. Inoculated spikes were covered with a plastic bag for 48 h to maintain high relative humidity. Disease symptoms were assessed at 7, 14, and 21 days post-inoculation (dpi), by calculating the percentage number of visually diseased florets (NDF) out of the total number of florets per spike. Differences in disease severity among genotypes were estimated by means of NDF ± SE (standard error) of 8–10 plants/genotype (4 each for R5 and R112, pooled) and by one-way analysis of variance (ANOVA). Seed number and weight (thousand grain weight, TGW) were assessed for inoculated and non-inoculated spikes of the same infected plants, and the differences among genotypes assessed as described above for disease severity. The same seeds were also used to extract wholemeal flour and determine the DON content (see below).

#### FCR: Seedling Inoculations With *F. culmorum*

Homozygous BC_2_F_3_ plants from the progeny of one of the tetraploid 7EL-7el_1_L HOM+, FHB resistant recombinants (R69-9/R112 cross derivatives), as well as of sib HOM− plants and of cv. Simeto as controls, were used in two independent infection experiments with *F. culmorum*. Twenty plants per genotype were included in each experiment. Seeds were surface sterilized with sodium hypochlorite (0.5% vol/vol) for 20 min and then rinsed thoroughly in sterile water. Seedlings were individually grown in 5 × 5 × 5-cm pots and arranged in plastic trays and maintained at the same light and temperatures regimes as described for the FHB assay throughout the experiments. *F. culmorum* strain UK99 macroconidia were produced by fungal culture on SNA medium and harvested by washing the culture surface with 2 ml sterile water (Urban et al., 2002). The inoculum solution contained 2 × 10^6^ ml^−1^ conidia (Beccari et al., 2011) and 0.05% Tween 20. As described in Ceoloni et al. (2017a), the inoculation procedure consisted of evenly spreading (with a small paintbrush) 20 μl of conidia suspension on the stem base leaf sheaths of plantlets at the first-leaf stage. Trays with inoculated plants were covered with a plastic film for 48 h to maintain high humidity conditions. Disease symptoms were assessed at 7, 11, 14, 18, and 21 days post-inoculation (dpi) measuring two parameters on the infected tissue: symptom extension (SE; cm) and browning index (BI, visual rating of the degree of extension of necrosis, as indicated by brown discoloration, based on a five-point scale: 0, symptomless; 1, slightly necrotic; 2, moderately necrotic; 3, severely necrotic; 4, completely necrotic). The final score, indicated as disease index (DI), was determined as SE × BI (Beccari et al., 2011). DI values, expressed as means ± SE of 20 plants per genotype and per experiment at each time-point, were subjected to two-way ANOVA.

#### Quantification of DON

DON content was determined in wholemeal flour of kernels produced by plants subjected to *F. graminearum* infection. Extraction and analytical procedures were performed as described in Mandalà et al. (2019). Briefly, the metabolite was extracted from 100 mg wholemeal flour dissolved in 400 µl of 86:14 acetonitrile:water (v/v) solution by prolonged shaking (24 h, 180 rpm, 4°C). After centrifugation, supernatants were injected into a UHPLC system (Ultimate 3000, Thermo) and run in positive ion mode. A Reprosil C18 column (2.0 mm × 150 mm, 2.5 μm—Dr. Maisch, Germany) was used for metabolite separation. The UHPLC system was coupled online with a mass spectrometer Q Exactive (Thermo) scanning in full MS mode (2 μ scans) at 70,000 resolution in the 60 to 1,000 m/z range. Data files were processed by MAVEN.52 (http://genomics-pubs.princeton.edu/mzroll/) upon conversion of raw files into mzXML format through MassMatrix (Cleveland, OH). Standard curves were obtained with six calibration points (2mg–0.00002 mg) of DON analytical standard (Romer Labs). To assess the effect of presence *vs*. absence of the *Fhb-7EL* QTL on DON content, each of the three *Fhb-7EL* carriers (R74-10/R112, R74-10/R5 and R69-9/R112 HOM+ derivatives) and non-carrier (bulked HOM− segregates, bulked R112+R5, and Simeto) genotypes was considered as a biological replicate. Bulks were a necessary option, due to limited amount of flour extracted from shriveled seeds of heavily diseased genotypes. For each replicate, seeds from all infected spikes were used to produce a single flour sample, from which three technical replicates were obtained. Values of all biological × technical replicates were analyzed by analysis of co-variance (ANCOVA), which, better than ANOVA, could eliminate the undesirable variable represented by genetic background heterogeneity across genotypes.

### Evaluation of Yield-Related and Quality Traits

Homozygous durum wheat BC_2_F_3–4_ recombinant plants (HOM+) from the R74-10/R112/2*Simeto and R69-9/R112/2*Simeto cross combinations, carrying different amounts of 7EL chromatin including the *Fhb-7EL* QTL, stacked into the same 7el_1_L segment of R112-7AL arm (see **Figure 1**), were field grown for 2 years (2017–18 and 2018–19 seasons) and in one locality (Viterbo, Central Italy, University of Tuscia Experimental Station), alongside sib plants of null segregates (HOM−) from the same progeny, as well as Simeto plants. In both seasons, plants were grown under common cultural practices and no fungicide application. In the 1^st^ season, BC_2_F_3_ plants were organized in randomized, triplicate rows (1 m long), at a 25-cm distance between rows and 10-cm distance along the row. In the 2^nd^ experimental year, the trial consisted of spike rows of BC_2_F_4_ selections of each HOM+ and HOM− genotype and of cv. Simeto. On separate plants (1^st^ year), data were collected for spike number/plant (SNP), grain number/plant (GNP), TGW, grain yield/plant (GYP), plant height (PH), days to heading (HD), and spike traits, including grain number/spike (GNS), grain yield/spike (GYS), spikelet number/spike (SPN), grain number/spikelet (GNSP), and spike fertility index (SFI). SFI, indicating the ability of the plant to set seeds in relation to the spike biomass, was calculated as the ratio between GNS and weight of spike chaff (g) of mature and oven-dried (48 h at 65°C) spikes. For each parameter, values from 20 to 30 plants per genotype, expressed as means ± SE, were subjected to one-way ANOVA. In the 2^nd^ year trial, besides PH and HD average values/row, the same spike traits mentioned above were analyzed on 25 spikes/genotype (five from each of five rows).

Harvested seed from BC_2_F_4_ plant rows was milled into semolina to measure the yellow index (YI) of contrasting genotypes for *Psy1* alleles. Using the reflectance colorimeter CR-400 Chroma Meter (Minolta), absolute measurements for L* (lightness), a* (red-green chromaticity), and b* (yellow-blue chromaticity) coordinates in the Munsell color system were taken using D65 lightning (reviewed in Ficco et al., 2014). The b* parameter, representing the variation in semolina YI, is known to be highly correlated with yellow pigment content (YPC) of whole-meal flour extracts (Ravel et al., 2013; Ficco et al., 2014). Semolina samples, each analyzed in triplicate (technical replicates), derived from seeds of three plants of HOM+ and HOM− sister lines/genotype and of Simeto.

As to leaf rust evaluation, aimed at confirming the efficacy of *Lr19*-based resistance, accurate scoring of disease severity was carried out in the 2018–19 season. A commonly used double-digit scale was adopted, in which the first digit indicates the rise of the disease, from the 1^st^ leaf (1) to spike (9; typically 8 = flag-leaf for leaf rust), with 0 = no disease, and the second digit corresponds to a one-value percentage of the average infection intensity on the leaf area (e.g., 3 = 30%), based on the modified Cobb scale (Peterson et al., 1948).

### Statistical Analyses

ANOVA and ANCOVA were performed using SYSTAT12 Software (Systat Software Incorporated, San Jose, CA, USA). The variable parameter (i.e., percentage of diseased florets for FHB, DI for FCR, and quantification of DON content in flour, each of various agronomic parameters) was considered as a dependent factor against the independent factor “genotype” (G). Additionally, “replica” (R) was included as independent factor in the two-way ANOVA performed for FCR assessment, or as a covariate in the ANCOVA used for DON and YI assays. Three levels of significance (*P* < 0.05, *P* < 0.01, and *P* < 0.001) were considered for F values. When significant values were observed, a pairwise analysis was carried out by the Tukey Honestly Significant Difference test (Tukey test) at 0.95 confidence level.

## Results

### Meiotic Pairing Analysis

**Figure 2:**
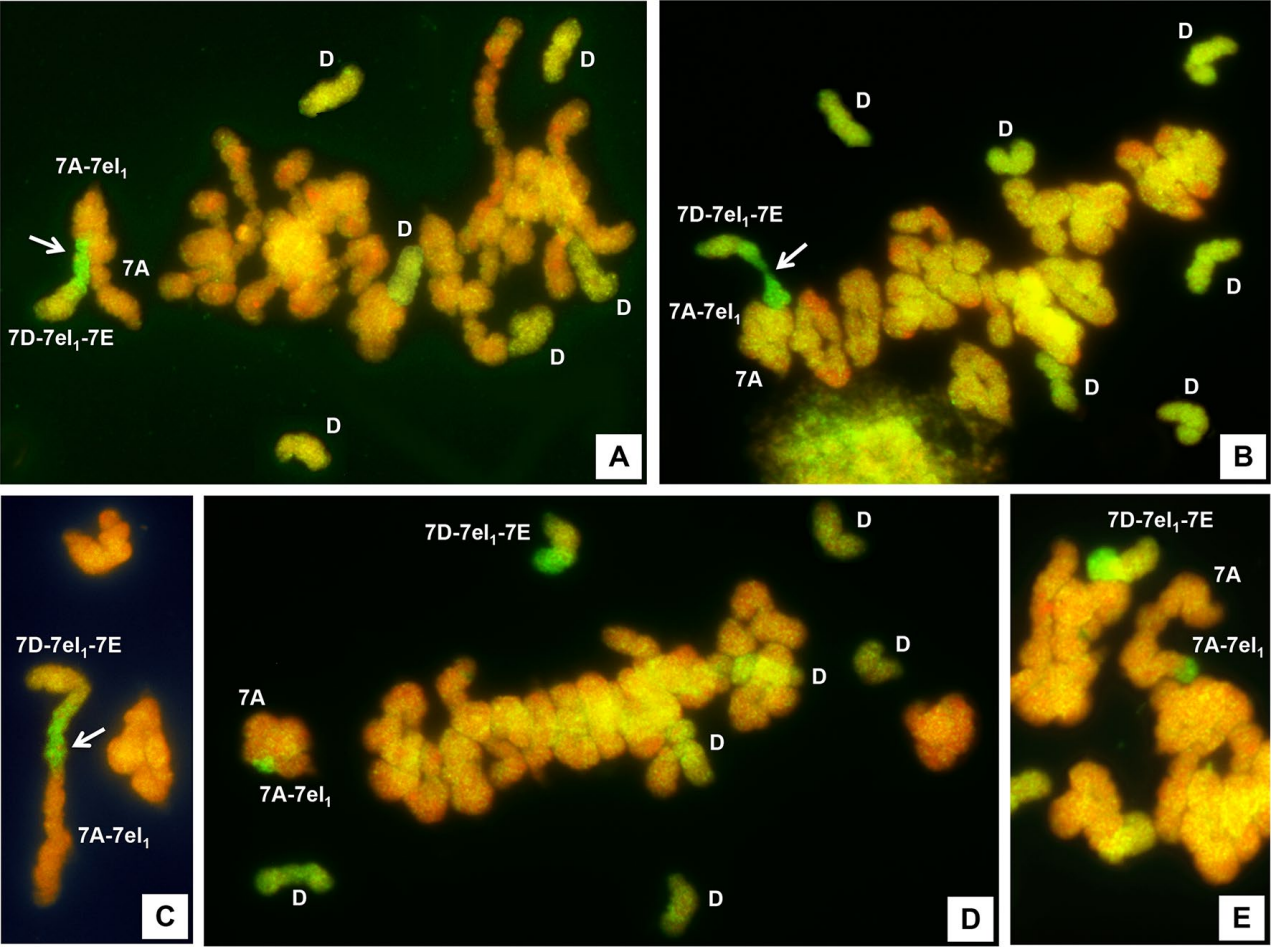
GISH of pollen mother cells (PMCs) at meiotic metaphase I stage of 5x F_1_ plants from the cross between R69-9 or R74-10 6x 7D-7el_1_-7E recombinant lines and R112 or R5 4x 7A-7el_1_ recombinant lines. Pairing in the 7el_1_L segments shared by the respective recombinant chromosomes (arrowed) is highlighted by the GISH site (bright green fluorescence) in the open **(A)** or closed **(B)** trivalent, and in the rod bivalent **(C)**. In **(D)** and **(E)**, the R69-9/R74-10 recombinant chromosome is unpaired (univalent), while a ring **(D)** or rod **(E)** bivalent is established between a complete 7A (from the 6x parent) and the 7A-7el_1_ chromosome from R112/R5. The greenish, univalent chromosomes from the D genome of the 6x parent are indicated **(D)** in plates **(A)**, **(B)** and **(D)**.

The ability to undergo meiotic metaphase I pairing by 7DS·7DL-7el_1_L/7EL chromosomes of R74-10 and R69-9 6x lines (bearing the *Fhb-7EL* QTL) and 7AS·7AL-7el_1_L chromosomes of R112 and R5 4x lines within their shared (homologous) 7el_1_L regions was assessed in PMCs of their 5x F_1_ plants processed by GISH. Because of the presence of a normal 7A from the 6x parent, homologous to the 7A of the durum parents at the short (S) arm level, a trivalent configuration was expected to occur if, in addition to 7AS-7AS pairing, that between the 7el_1_L homologous regions of R74-10/R69-9 and R112/R5 would have also taken place. This was in fact the type of association in which a GISH site at the level of a chiasmate region was observed in the largest majority of PMCs in all F_1_ types (**Figures 2A, B**; **Table 2**). Only a small percentage (ranging from 1.6 to 6.7) of PMCs showed the R74-10 or R69-9 chromosome paired with R112 or R5 in the form of a rod bivalent (**Figure 2C**), as a consequence of failure of 7AS-7AS pairing. Concerning the trivalent associations, these were prevailingly of the open type (**Figure 2A**), but a considerable proportion was of the closed type (**Figure 2D**), evidently resulting from formation of a second chiasma, proximal to that between 7el_1_L portions, between the 7AL arms. In PMCs where the critical chromosomes, carrying *Thinopyrum* spp. chromatin, were unpaired, the 7D-7el_1_-7E chromosome of either R69-9 or R74-10 was invariably observed as a univalent (**Figures 2D, E**). On the contrary, because of the presence in the same cells of a normal 7A (see above), the 7A-7el_1_ chromosome of R5 or R112 paired with the latter in over 95% of PMCs, mostly as ring bivalent (60% of cases) rather than as a rod bivalent, both associations clearly marked by the GISH hybridization site of the 7el_1_L segment of R5 or R112 (**Figures 2D, E**, respectively).

Overall, the total amount of 7el_1_L–7el_1_L pairing varied in proportion to the length of homologous 7el_1_L portion shared by the two parental chromosomes in each F_1_ type. Pairing frequency (*pf*) was higher in cross combinations involving the longer 7el_1_L segment of R112 than in those involving R5, and, concomitantly, in combinations where the shorter 7EL segment of R69-9 was involved compared to those including the R74-10 chromosome (**Figure 1**). As a result, higher *pf* were observed between R69-9 or R74-10 and R112 chromosomes (78.3 and 72.5%, respectively; **Table 2**), as compared with those involving R5 (60.7 and 42.2%, respectively).

**Table 2:** Meiotic metaphase I pairing behavior of 7D-7el_1_-7E and 7A-7el_1_ chromosomes in pollen mother cells (PMCs) of pentaploid hybrids from crosses between 6x recombinants (R74-10 or R69-9) and 4x recombinants (R112 or R5).

Cross combination (6x/4x)	No. PMCs	% 7el_1_-7el_1_ pairing	Mode of 7E-7el_1_ pairing (%)		
			Open trival.	Closed trival.	Rod bival.
R74-10/R112	68	72.5 ± 1.5	55.3 ± 4.2	38.5 ± 0.5	6.2 ± 3.1
R74-10/R5	91	42.2 ± 1.0	63.4 ± 1.9	30.0 ± 1.9	2.2 ± 2.2
R69-9/R112	96	78.3 ± 3.3	72.9 ± 0.4	25.5 ± 1.3	1.6 ± 1.0
R69-9/R5	64	60.7 ± 3.2	60.5 ± 7.2	32.8 ± 4.3	6.7 ± 3.8

Pairing figures are expressed as means ± standard errors; values concerning the percentage of 7el_1_-7el_1_ pairing derive from PMCs extracted from 2–3 plants/cross combination.

### Isolation of 7EL+7el_1_L Durum Wheat Recombinants

To isolate recombinant types within progeny of the crosses of cv. Simeto with the various 5x types (considered equivalent to BC_1_ to durum wheat of 6x parents; see Materials and Methods), marker-based genotyping was carried out (**Table 1**). In particular, BE405003 was useful at revealing presence of the associated *Fhb-7EL* QTL, and BE445653 or GWM344 confirmed the origin of the proximally adjacent region (7el_1_L for R69-9, 7EL for R74-10). Afterwards, a PCR assay for the further proximal segment enabled discrimination between parental and recombinant chromosomes, as well as between recombinant types bearing the 7EL+7el_1_L assembly on 7AL rather than on 7DL (**Figure 1**). Presence/absence of the 7DS marker GWM573 provided a further validation of both recombinant and parental genotypes. In 7A recombinants, the presence of 7el_1_L target genes, such as *Lr19* and *Psy1* (for the *Yp* trait; see Introduction), was confirmed by the respective markers (**Table 1**). As expected (see Introduction), none of the isolated recombinants showed dissociation of most distal loci with respect to the parental allelic makeup; this indicated, at least at the resolution level allowed by the markers used, that no homoeologous 7EL-7el_1_L recombination occurred.

A total of 38.3% of recombinant types were isolated in the BC_1_ to durum wheat (**Table 3**). The remaining genotypes were prevailingly of the parental type, either R69-9/R74-10 (P1, 24.8%) or R5/R112 (P2, 18.4%). A minor percentage was that of non-recombinant genotypes in which the P1 and P2 chromosomes, due to pairing failure (hence behaving as univalents at meiosis), underwent abnormal segregation, being eventually either both incorporated (P1+P2 types) or excluded (“7A only” types) from gametes. The relative percentage of these abnormal types was expectedly higher in progenies of cross combinations exhibiting the lowest pairing values, i.e., R74-10/R5 and R69-9/R5 (**Table 3**).

**Table 3:** Recombination frequency and genotypes isolated in the cross progeny to durum wheat cv. Simeto of pentaploid F_1_s (6x recombinants, R74-10 or R69-9 × 4x recombinants, R112 or R5).

F_1_ hybrid	Progeny types (29 < 2n < 32)								
	No. plants	Recombinants		Rec. frequency (%)		6x parental chromosome	4x parental chromosome	Co-presence	7A only
		7A	7D	TOT.	Gametic (7A)	R74-10 or R69-9 (P1)	R112 or R5 (P2)	P1 + P2	
R74-10/R112	21	4	7	52.4	36.4	3	5	2	–
R74-10/R5	39	2	9	28.2	18.2	6	9	6	7
R69-9/R112	36	4	13	47.2	23.5	16	2	–	1
R69-9/R5	45	4	11	33.3	26.7	10	10	6	4
Total	141	14	40			35	26	14	12
%	100	9.9	28.4			24.8	18.4	9.9	8.5

Recombination frequency (*rf*) resulting from exchanges within the 7el_1_L shared chromatin between R74-10/R69-9 and R112/R5 showed the expected trend from the *pf* values. Except for the R69-9/R5 *rf*, the other values exceeded the expected 50% of the respective *pf*, probably due to metaphase I observations providing an underestimate of actual (early prophase I) pairing events (see also Gennaro et al., 2012). The *rf* data confirmed the main contribution to 7el_1_L-7el_1_L pairing and crossover occurrence of the 5%-long segment differentiating R112 from R5, the highest values corresponding to F_1_s containing the R112 chromosome *vs*. those including R5 (**Table 3**).

In the progeny of all cross combinations, recombinant 7D chromosomes (and, to a lesser extent, parental 7D types) prevailed over recombinant 7As. Nonetheless, sufficient representatives of all 7A novel (7EL+7el_1_L) recombinant types were isolated, with chromosome numbers ranging from 2n = 29 to 2n = 32 (all other genotypes of the BC_1_ progeny to durum wheat of 5x F_1_s fell within the same range). A further backcross of selected plants (2n = 29, 30 or 31) to the same cv. Simeto brought most of them (over 80%) to the euploid (2n = 28) condition, hence to stabilization of 7A-7el_1_L-7EL recombinant genotypes in view of further analyses.

A first check of their stability, also aimed at obtaining homozygous recombinant (HOM+) and non-recombinant (HOM–) individuals, was carried out by screening the BC_2_F_2_ progeny of euploid recombinant plants by a single codominant marker, namely, BE445653 in the case of R69-9 derivatives (7el_1_ and 7A alleles; **Figure 1**) and GWM344 for R74-10 derivatives (7E and 7A alleles; **Figure 1**). Such markers allowed discrimination of HOM+, HOM–, and heterozygous (HET) segregates, whose ratio was compared with the expected 1:2:1 for normal segregation (**Table 4**). The χ^2^ test was in all cases associated with probability (*P*) levels indicative of normal gametic transmission (> 5%), although *P* values were higher for progenies involving the R69-9 chromosome than for those involving R74-10.

**Table 4:** Segregation ratios of novel 7A recombinant chromosomes in tetraploid BC_2_F_2_ progenies from crosses to durum wheat (R112 or R5/2*cv. Simeto) of 6x recombinants (R74-10 or R69-9).

Cross combination (6x/4x)	No. BC_2_F_2_ plants	Segregation			**χ**^2^ (1:2:1)	*P* value (%)
		HOM+	HET	HOM−		
R74-10/R112	61	9	31	21	4.74	9.4
R74-10/R5	54	6	31	17	5.67	5.9
R69-9/R112	60	14	32	14	0.27	87.4
R69-9/R5	88	23	39	26	1.34	51.0

### Effects of *Fusarium* spp. Infections in the Presence vs. Absence of the *Fhb-7EL* QTL

#### Reaction to Spike Inoculation With *F. graminearum*

**Figure 3:**
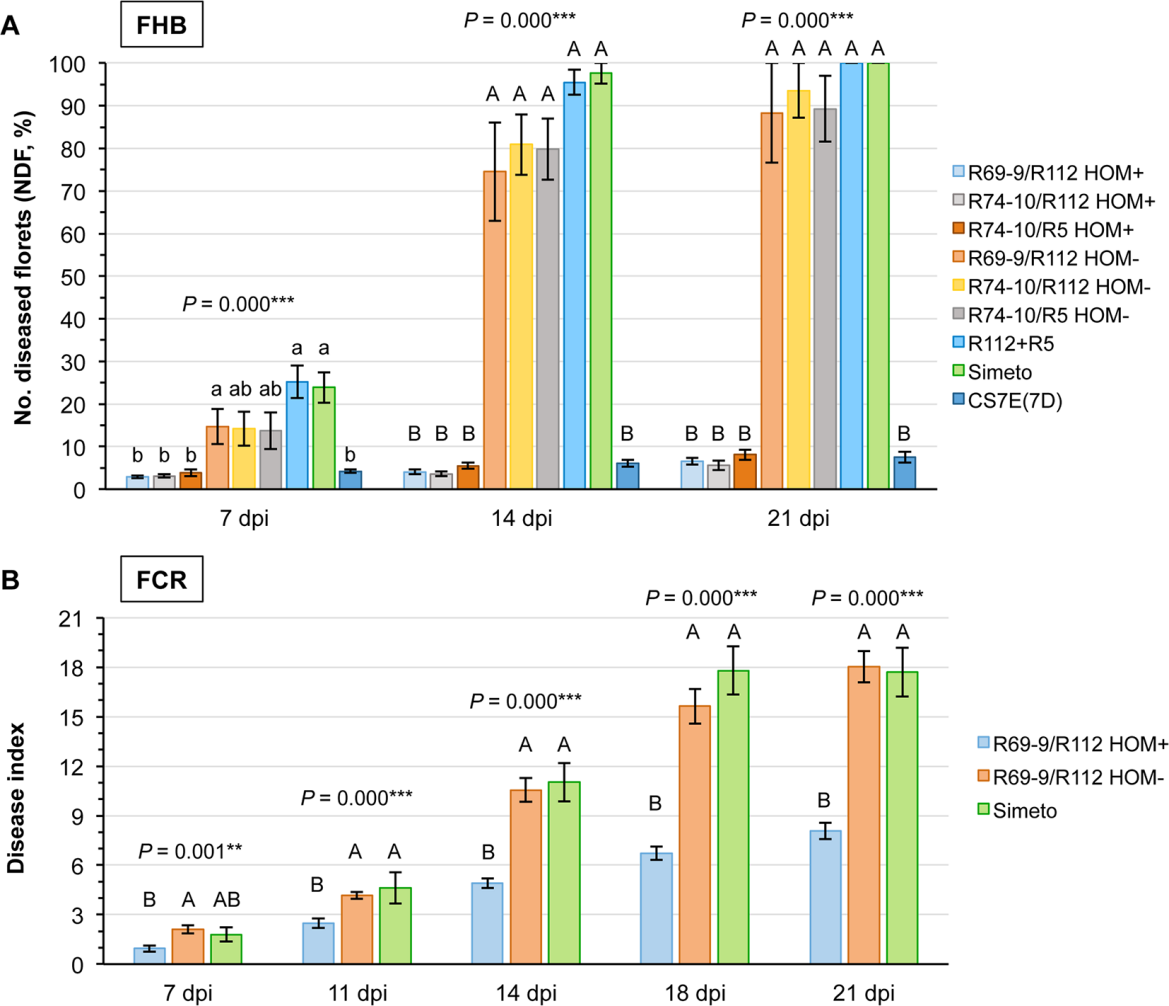
Evaluation of FHB **(A)** and FCR **(B)** symptom development at different time-points following inoculation (dpi = days post-inoculation) in durum wheat homozygous carriers (HOM+) and non-carriers (HOM− segregates and cv. Simeto) of the *Fhb-7EL* QTL. The hexaploid CS7E(7D) original donor line of *Fhb-7EL* is included as FHB resistant control in **(A)**. Data at all time points were subjected to ANOVA analysis, and significant F values indicated by **P < 0.01 and ***P < 0.001, respectively.

**Figure 4:**
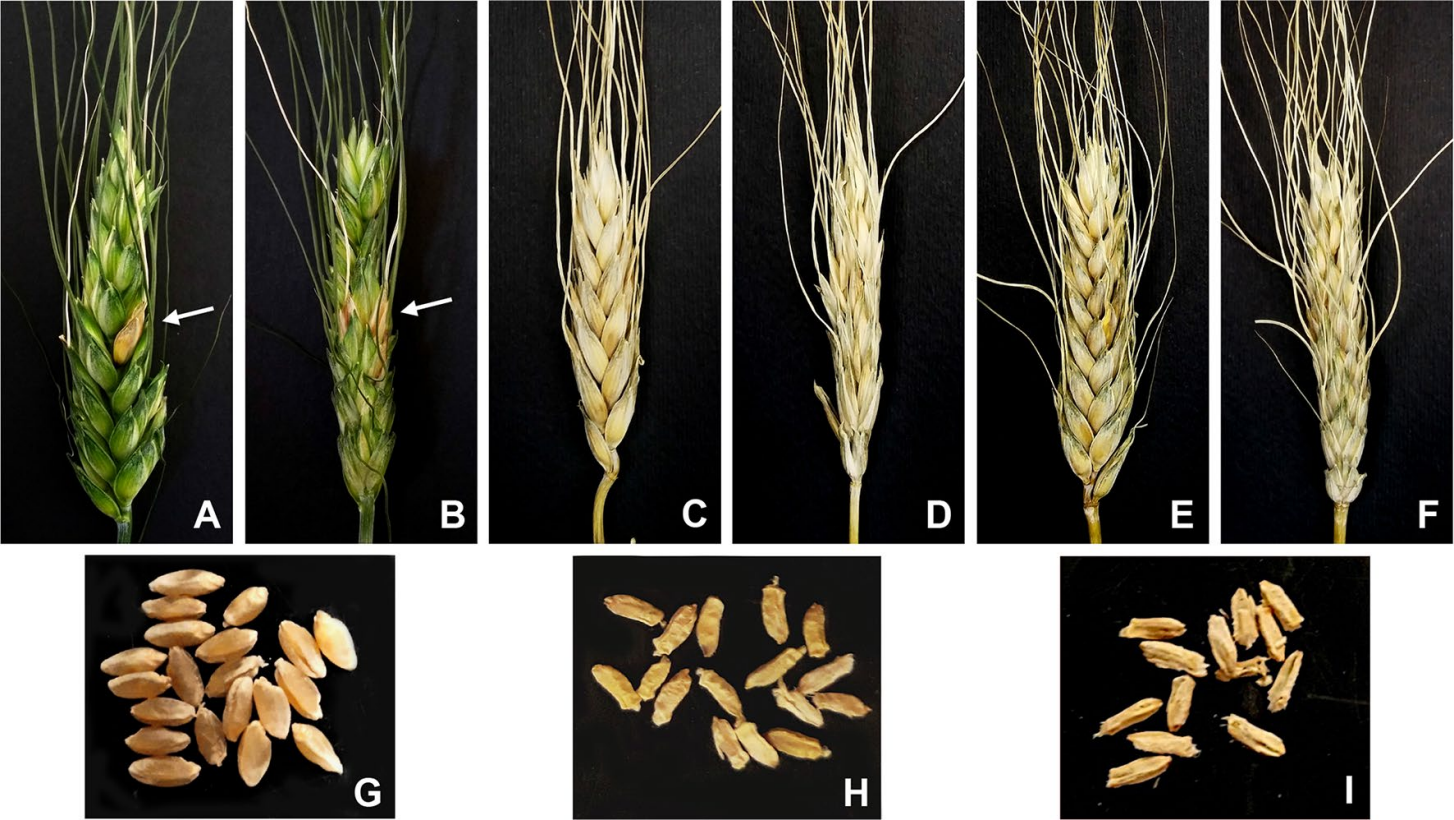
Phenotypes of *F. graminearum* inoculated spikes (21 dpi) and of corresponding harvested seeds of durum wheat-*Thinopyrum* spp. 7A-7el_1_L-7EL recombinant lines and control lines. **(A-B)** FHB resistant (*Fhb-7EL*+) recombinant (HOM+), with arrows pointing at the diseased floret(s)/spikelet(s); fully diseased spikes of HOM− segregates in the same BC_2_F_2_ progenies from the cross to durum wheat of 5x F_1_s **(C-D)** and of durum wheat cv. Simeto **(E-F)**. Mature seeds of HOM+ **(G)**, HOM− **(H)**, and Simeto **(I)** genotypes show a sharp difference in plumpness.

Progression of infection through the three time-points (7, 14, and 21 dpi) following single-floret inoculation with *F. graminearum* unequivocally discriminated carriers [HOM+ segregates of BC_2_F_2_ progenies from the cross to durum wheat of 5x F_1_s, see above, and the CS7E(7D) substitution line] from non-carriers (HOM– segregates, R112+R5, and Simeto) of the *Fhb-7EL* QTL (**Figure 3A**). Regarding the former group, number of diseased florets (NDF) in the HOM+ plants of three tetraploid recombinant genotypes did not exceed 8% even at 21 dpi, with values at this time-point being not significantly different among the three cross combinations. This NDF value was only slightly superior than that recorded at 14 dpi, which in turn was only somewhat higher than that at 7 dpi of the corresponding genotypes. This trend is indicative of a very minor progression of the FHB disease from the inoculation time and site (**Figures 4A, B**). Considering altogether the reaction of 4x resistant (*Fhb-7EL*+) *vs*. susceptible (*Fhb-7EL*–) genotypes, the reduction in FHB severity in the former amounted to nearly 93%.

As indicated by the Tukey test ranking (**Figure 3A**), values of NDF expressed by 4x HOM+ segregates at all time-points were not significantly different from those of the 6x CS7E(7D) FHB resistant control. By contrast, in all genotypes known to lack the *Fhb-7EL* QTL infection progress was much faster, reaching the majority and even 100% of florets and spikelets of the inoculated spike between 14 and 21 dpi (**Figures 3A** and **4C–F**).

The conspicuous difference in FHB severity among 4x genotypes sharing a similar background, i.e., that of cv. Simeto, revealed by the NDF parameter was likewise obvious when the seed set and the grain weight of inoculated and non-inoculated spikes of the same plants were measured (**Table 5**). The Tukey test ranking showed that seed set of inoculated spikes of all genotypes carrying the *Fhb-7EL* QTL was significantly greater than that of genotypes lacking the QTL (average 23.4 seeds/spike *vs*. 6.2, respectively), corresponding to 73.5% reduction in seed number/spike in the susceptible plants. In parallel, average TGW calculated for inoculated spikes of FHB resistant 4x genotypes was 33.8 g, in sharp contrast with the 6.7 g average TGW of susceptible genotypes (over 80% reduction). In fact, conspicuous shrivelling (**Figures 4H, I**) was consistently observed in the few seeds occasionally produced by severely diseased spikes, whereas no significant alteration of plumpness and weight was detected in seeds of infected spikes of FHB resistant genotypes (**Figure 4G**).

**Table 5:** Effects of *F. graminearum* infection on fertility traits of mature spikes of durum wheat homozygous carriers (HOM+) and non-carriers (HOM−) of the *Fhb-7EL* QTL.

Genotype	Fhb-7EL	Inoculated spike		Remaining spikes	
		No. seeds	TGW	No. seeds	TGW
R69-9/R112	HOM+	24.0 ± 2.8 A	33.8 ± 2.1 A	23.8 ± 1.4	33.2 ± 2.0
	HOM–	4.6 ± 2.1 B	3.5 ± 1.9 B	21.5 ± 2.4	34.8 ± 3.0
R74-10/R112	HOM+	25.3 ± 1.2 A	32.9 ± 1.3 A	23.5 ± 2.2	34.3 ± 1.9
	HOM–	8.2 ± 2.3 B	6.8 ± 1.0 B	22.1 ± 1.5	32.4 ± 2.6
R74-10/R5	HOM+	21.0 ± 2.1 A	34.6 ± 2.6 A	21.7 ± 1.0	38.3 ± 2.6
	HOM–	2.5 ± 1.2 B	12.2 ± 1.3 B	21.2 ± 2.7	36.8 ± 2.6
R112+R5	HOM–	10.1 ± 1.8 B	5.7 ± 1.1 B	23.0 ± 1.3	38.9 ± 2.1
Simeto	HOM–	5.6 ± 1.1 B	5.1 ± 0.9 B	28.4 ± 1.1	35.9 ± 2.9
ANOVA *P*-value		0.000***	0.000***	0.062	0.439

Values are expressed as means ± standard errors; letters indicate ranking of the Tukey test at P < 0.01; *** indicates significant F values at P < 0.001. The first three sets of genotypes (HOM+/HOM–) are BC_2_F_2_ segregates from the cross of R69-9 or R74-10 with T. durum cv. Simeto background (see text).

That the defects in grain number and weight were ascribable to the fungal attack is demonstrated by the GNS and TWG values of the remaining (non-inoculated) spikes of the same plants (**Table 5**). Both R112+R5 and Simeto showed a normal seed set in such spikes, which was not significantly different from that of non-inoculated (or even inoculated) spikes of FHB resistant genotypes (average for all genotypes around 23 seeds/spike). Concomitantly, TGW was very similar among genotypes when non-infected spikes were compared (**Table 5**).

UHPLC-MS analyses were performed to quantify the content of DON mycotoxin in flour extracted from mature grains of the 4x FHB resistant recombinants (*Fhb-7EL* HOM+) and susceptible controls (*Fhb-7EL* HOM–). The 3 resistant recombinants, taken as biological replicates of the *Fhb-7EL*+ condition, showed an average value of 0.67 ppm, more than 800 times lower than the 547.4 ppm mean figure of the 3 genotypes representing the *Fhb-7EL*– condition (**Table 6**). No appreciable difference was detected among HOM+ lines, whereas DON content of different genotypes lacking *Fhb-7EL* varied. The lower DON content exhibited by HOM– segregates relative to R112+R5 and Simeto (with a similar, though not significant trend present in FHB infection data; see **Figure 3A**) is probably due to minor FHB resistance QTL in their background (including CS, from the original donor line of the *Fhb-7EL* QTL; see Materials and Methods and Ceoloni et al., 2017a).

**Table 6:** Deoxynivalenol (DON) content in wholemeal flour from seeds of infected spikes of carrier (HOM+) and non-carrier (HOM–) genotypes of the *Fhb-7EL* QTL.

Genotype	Fhb-7EL	DON (ppm)	
R69-9/R112	HOM+	0.47 ± 0.1 c	
R74-10/R112	HOM+	0.66 ± 0.0 c	0.673 ± 0.1
R74-10/R5	HOM+	0.89 ± 0.1 c	
Null segregates	HOM–	176.64 ± 19.3 b	
R112+R5	HOM–	685.97 ± 44.9 a	547.4 ± 96.8
Simeto	HOM–	779.73 ± 69.5 a	
ANCOVA *P*-value		0.000***	0.000***

DON values are expressed as means ± standard errors; those regarding individual genotypes are followed by letters corresponding to ranking of the Tukey test at P < 0.05; values reported in the last column refer to the 3 HOM+ and the 3 HOM– genotypes, taken as biological replicates (see Materials and Methods). *** indicates significant F values at P < 0.001. Genotypes are the same as described in **Table 5**.

#### Reaction to Spike Inoculation With *F. culmorum*

**Figure 5:**
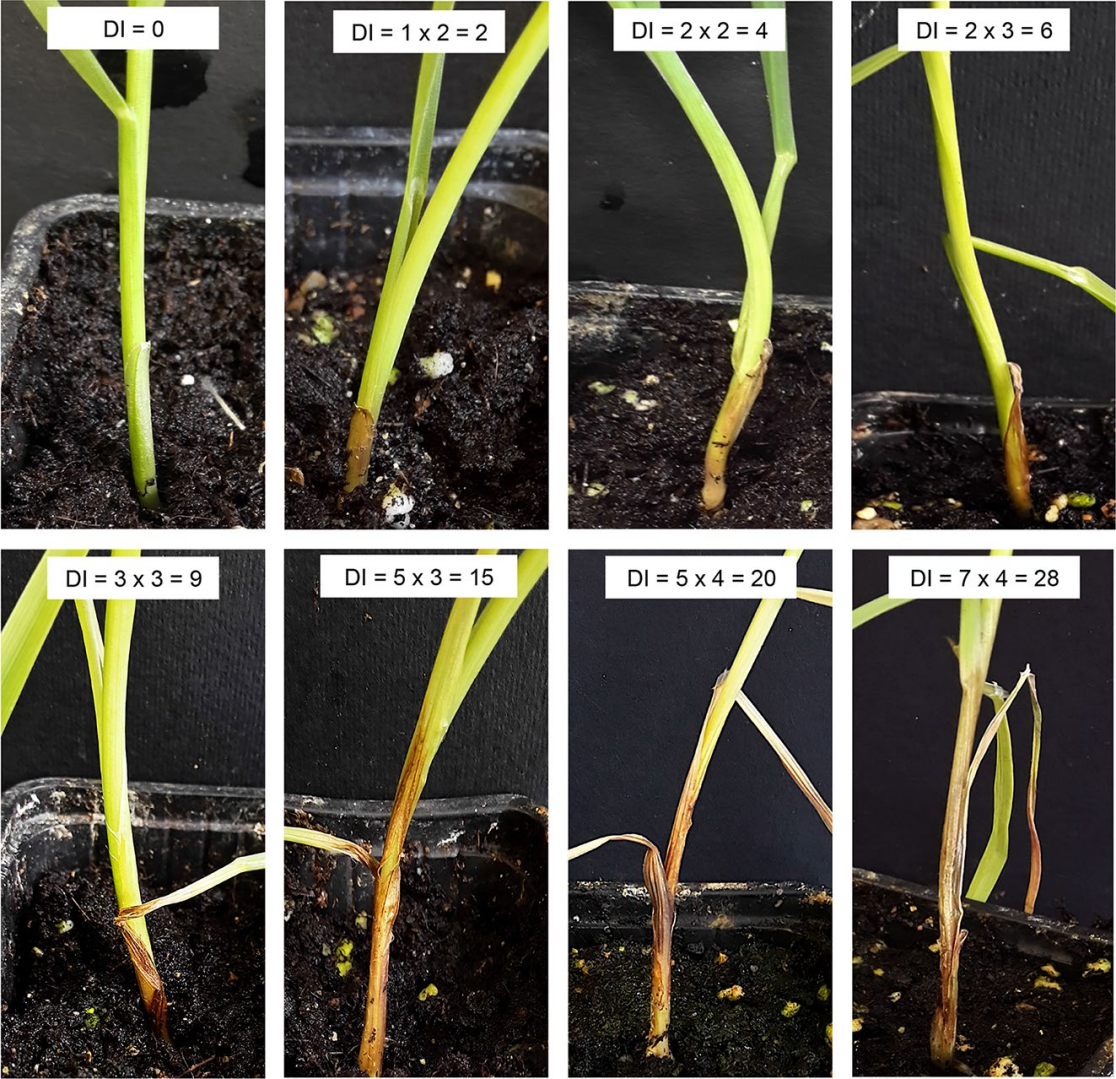
Examples of FCR disease symptoms recorded on seedling stem base leaf sheath of R69-9/R112/2*Simeto derivatives. DI, disease index = SE (symptom extension, cm) x BI (browning index; see Materials and Methods for further details.

To monitor visible disease progress accurately, the time-course of the present FCR infection assay, conducted on seedlings of tetraploid *Fhb-7EL* HOM+, FHB resistant recombinant plants (R69-9/R112 cross derivatives), as well as on FHB susceptible control plants (HOM− sibs and cv. Simeto), was extended of 1 week with respect to a previous experiment (Ceoloni et al., 2017a), with five time-points between inoculation and 21 dpi (**Figure 3B**). The DI values of HOM− (*Fhb-7EL*−) and Simeto plants largely overlapped throughout the time-points, collectively showing a highly significant difference *vs*. *Fhb-7EL*-bearing plants, especially from 11 dpi onward (**Figure 3B**). From this time-point, FCR symptoms increased rapidly in seedlings of genotypes lacking *Fhb-7EL*, reaching average DI values of about 11 at 14 dpi and 17-18 at 18-21 dpi, with peaks of up to 28 recorded (**Figure 5**). By contrast, a much slower progression was observed in *Fhb-7EL* HOM+ plants, exhibiting a maximum DI of around 8 (21 dpi), characterized by limited SE and brown discoloration of the infected tissue (**Figures 3B** and **5**). As a whole, during 14 to 21 dpi, FCR symptom severity was consistently reduced by 55–60% in *Fhb-7EL*+ compared with *Fhb-7EL*− plants. No major disease intensification was observed on the former plants beyond the 21 dpi assessment, while several *Fhb-7EL*− seedlings withered completely (not shown).

### Agronomic and Quality Features of Novel Recombinant Genotypes

Sufficient seed was available to run preliminary tests of performance under field conditions of two of the newly obtained durum wheat recombinant lines, deriving from the R69-9/R112 and R74-10/R112 cross combinations. The small-scale trials (see *Materials and Methods*) included F_3−4_ HOM+ and HOM− sib plants from R74-10 and R69-9/R112/2*Simeto crosses, as well as the recurrent cv. Simeto. In both experimental years, no major penalty on yield-related traits was found to be associated with presence of alien segments. In the 1^st^ season (2017–18), yield-related traits such as spike number, grain number, and grain yield per plant (SNP, GNP, and GYP; **Table 7A**) gave higher values in both HOM+ lines *vs*. their respective HOM− sibs and Simeto, although ANOVA showed differences to be significant only for SNP. As to spike traits, SPN differed significantly among genotypes, though not in a clear-cut relation with presence/absence of any alien introgression. GNS and SFI also showed some variation among genotypes (**Table 7A**). However, for most spike traits, differences became highly significant and genotype-dependent in the 2018–19 season (**Table 7B**). In particular, both recombinant (HOM+) genotypes outperformed their HOM− sibs for GNS and GNSP. A positive background effect was evident in HOM+ and HOM− R69-9/R112 selections, resulting in significantly higher SPN and GYS values compared with all other genotypes (**Table 7B**).

**Table 7:** Spike and plant traits of field-grown homozygous (HOM+) recombinants (R74-10 or R69-9/R112/2*Simeto F_3-4_ derivatives) compared to corresponding HOM– segregates and to the recurrent cv. Simeto.

Trait	R74-10/R112 HOM+	R74-10/R112 HOM–	R69-9/R112 HOM+	R69-9/R112 HOM–	Simeto	ANOVA *P* value
**A. 2017–18**						
SNP	7.6 ± 1.0	5.1 ± 0.5	6.2 ± 0.4	5.3 ± 0.6	4.9 ± 0.6	0.038*
GNP	231.4 ± 32.7	176.0 ± 15.6	184.0 ± 13.2	162.7 ± 14.0	148.0 ± 18.7	0.087
GYP	11.1 ± 1.5	8.8 ± 0.8	8.7 ± 0.7	7.6 ± 0.7	7.6 ± 1.1	0.128
GNS	47.6 ± 2.2	46.1 ± 2.3	52.1 ± 1.6	53.5 ± 2.3	45.5 ± 1.8	0.018*
SPN	17.7 ± 0.4 bc	16.8 ± 0.4 c	18.3 ± 0.3 b	19.7 ± 0.3 a	16.9 ± 0.3 c	0.000***
GNSP	2.7 ± 0.1	2.7 ± 0.1	2.8 ± 0.1	2.7 ± 0.1	2.7 ± 0.1	0.601
SFI	56.4 ± 2.0	53.1 ± 2.2	57.1 ± 1.2	61.1 ± 1.5	54.2 ± 1.7	0.038*
TGW	48.2 ± 0.7	50.2 ± 1.2	47.3 ± 0.8	46.9 ± 1.3	50.5 ± 1.4	0.101
GYS	2.4 ± 0.1	2.6 ± 0.1	2.6 ± 0.1	2.7 ± 0.1	2.5 ± 0.1	0.401
HD	117.3 ± 0.3	117.6 ± 0.4	118.7 ± 0.5	118.4 ± 0.3	119.0 ± 0.6	0.055
PH	70.1 ± 1.2 bc	68.3 ± 1.3 c	72.5 ± 0.8 ab	68.2 ± 1.4 c	75.1 ± 1.1 a	0.000***
**B. 2018–19**						
GNS	49.5 ± 1.2 c	44.2 ± 1.5 d	61.2 ± 1.0 a	54.9 ± 1.3 b	49.7 ± 0.9 c	0.000***
SPN	17.9 ± 0.2 b	17.3 ± 0.3 bc	19.2 ± 0.2 a	20.0 ± 0.2 a	16.9 ± 0.2 c	0.000***
GNSP	2.8 ± 0.1 b	2.5 ± 0.0 c	3.2 ± 0.1 a	2.7 ± 0.1 bc	2.9 ± 0.0 b	0.000***
SFI	46.5 ± 1.2 b	50.7 ± 1.5 ab	55.3 ± 1.3 a	52.7 ± 1.7 a	53.0 ± 1.4 a	0.001**
TGW	53.9 ± 0.8	52.2 ± 1.5	49.6 ± 1.0	51.8 ± 0.2	52.6 ± 0.7	0.063
GYS	2.7 ± 0.1 bc	2.3 ± 0.1 b	3.0 ± 0.1 a	2.8 ± 0.1 ab	2.6 ± 0.1 c	0.000***
HD	112.4 ± 0.5 b	115.3 ± 0.5 a	115.4 ± 0.2 a	114.5 ± 0.6 a	114.5 ± 0.3 a	0.001**
PH	88.9 ± 1.3 ab	81.7 ± 1.8 c	89.9 ± 1.2 a	83.7 ± 1.6 bc ab	87.5 ± 1.6 abc	0.001**
YI	22.1 ± 0.5 B	22.2 ± 0.4 B	31.4 ± 0.5 A	23.0 ± 0.3 B	22.9 ± 0.2 B	0.000***
LR	0	8-5	0	7-6	8-5	–

SNP, spike number/plant; GNP, grain number/plant; GYP, grain yield/plant; GNS, grain number/spike; GYS, grain yield/spike; SPN, spikelet number/spike; GNSP, grain number/spikelet; SFI, spike fertility index; TGW, thousand grain weight; HD, days to heading (from January 1^st^); PH, plant height; YI, yellow index; LR, leaf rust. Except for LR, trait values are given as means ± standard errors; in case of significant differences among genotypes, these are followed by letters corresponding to ranking of the Tukey test at P < 0.01 (capital) and P < 0.05 (lower case) levels. *, **, *** indicate significant F values at P < 0.05, P < 0.01, and P < 0.001, respectively.

A mild leaf rust attack occurred during the 2017–18 season. Despite this, the pathogen produced visible pustules on HOM− and Simeto plants, while leaving HOM+ sibs, carriers of the *Lr19* gene within their 7el_1_L segments (**Figure 1**), totally rust-free. In the 2018–19 season, a stronger natural infection took place, which allowed clearer discrimination among genotypes. As values were rather consistent within each genotype, a single double-digit record has been reported/genotype (**Table 7B**), corresponding to the peak-time of the disease progress. No disease symptom was recorded on the novel recombinant types, whereas in their HOM− sibs and Simeto the infection reached the flag leaf (score “8”) or the penultimate leaf (score “7”), with pustules covering 50–60% of the leaf area (“5” and “6” second digit in **Table 7B**). In the test environment, there was no evidence of FHB presence in the 1^st^ season, and only a sporadic appearance in the 2^nd^ one, which, however, did not involve any of the materials under assay. In the absence of stem rust epidemics, the presence of the 7el_1_L-linked *Sr25*, to be excluded in R74-10 derivatives on the basis of mapping data (Ceoloni et al., 2017a), remains to be ascertained in R69-9 derivatives.

A highly significant difference was revealed by the colorimetric test for the semolina YI of genotypes, alternatively carrying the *Psy1-7el* *_1_* *L* (R69-9/R112 HOM+) or the *Psy1-7EL *(R74-10/R112 HOM+) allele in place of a *Psy1-7AL* allele (HOM− lines and Simeto). Presence of *Psy1-7el* *_1_* *L* from *Th. ponticum* determined a 37–42% increase compared with all other genotypes, while the *Th. elongatum* *Psy1-7EL* allele had no incremental effect *vs*. the *T. durum* 7AL resident allele (**Table 7B**).

## Discussion

### Effectiveness of the Transfer Strategy

In the present study, we successfully exploited meiotic recombination, confined to a homologous *Th. ponticum* 7el_1_ chromosome segment shared by two selected pairing partners, to create a new pyramid of positive alien genes/QTL, including a potent *Fusarium* spp. resistance locus, into durum wheat chromosome 7A. Previous studies (e.g., Ceoloni and Jauhar, 2006 for review) have widely demonstrated that homoeologous pairing-based wheat-alien chromosome engineering carried out at the tetraploid level, i.e., with durum wheat as the primary recipient crop species, leads to much less success than when hexaploid bread wheat is targeted. Besides the overall reduced tolerance to chromosome manipulations associated to the lower ploidy level, a further limiting factor is represented by closer affinity between certain alien genomes, such as those of some widely exploited *Thinopyrum* species, to the wheat D genome, compared with A and B genomes (see, e.g., Forte et al., 2014; Ceoloni et al., 2017a, and references therein). This results in excellent performance of corresponding recombinant products even in the presence of sizable introgressions. The latter case is well exemplified by the bread wheat T4 translocation line, widely used in breeding (reviewed in Ceoloni et al., 2015).

In the present work, the recently obtained T4 derivatives R74-10 and R69-9 (Ceoloni et al., 2017a), containing the *Fhb-7EL* resistance QTL at the distal end of their 7DL-7el_1_L arm (i.e., 7DL-7el_1_L-7EL), were selected to transfer *Fhb-7EL* into the 7AL-7el_1_L durum wheat recombinant chromosomes of R5 and R112 lines (Ceoloni et al., 2005). The choice of parental recombinant types was inherent to their structure; in fact, they could provide the physical basis for spontaneous pairing and recombination to occur in the common 7el_1_L portion to their otherwise homoeologous target chromosomes, both in the most distal end of the same arm (7EL *vs*. 7el_1_L) and in the remaining portions (7D *vs*. 7A; see **Figure 1**). Even the limited extension of the shared 7el_1_L segment in all R74-10/R69-9 with R5/R112 chromosome combinations turned out to be sufficient to recover the novel 7EL+7el_1_L recombinant types at a relatively high rate (**Table 3**). Although never tested previously in the same chromosomal and genomic context as presented here, this result was not totally unexpected. As a matter of fact, in wheat and in many other species, the distribution of pairing and crossover (CO) events follows a telomere-to-centromere gradient, with concentration of such events in the distal half or even less of the physical arm length, both between homologous and homoeologous chromosomes (Lukaszewski and Curtis, 1993; Lukaszewski, 1995; Saintenac et al., 2009, Saintenac et al., 2011; Higgins et al., 2012; Darrier et al., 2017; Jordan et al., 2018). In general terms, the location of the shared 7el_1_L segment in the different cross combinations could be considered to fall within the high recombinogenic chromosomal space. Moreover, the virtual absence of pairing in the homoeologous most terminal 7EL-7el_1_L regions in all 5x F_1_s, accompanied by further interruption of homology in the more proximal arm portions of the same pairing partners, evidently favored pairing and recombination in the only 7el_1_L homologous interval available to the respective parental recombinant chromosomes. In this respect, several examples have demonstrated dramatic effects on pairing and CO frequency and distribution as a result of regional differences in the structure of potential pairing partners, mainly at the telomeric ends (see, e.g., Lukaszewski et al., 2004 and references therein). Furthermore, the current results indicate a particularly high propensity for pairing and CO of the roughly 5% 7el_1_L chromatin differentiating R112 from R5 (see crosses with R112 of either R69-9 or R74-10 in **Table 3**), even irrespective of the somewhat wider space in the more distal vicinity, as in corresponding crosses with R5. Interestingly, this is the interval within which 7AL-7el_1_L *ph1*-induced homoeologous pairing gave rise to three recombination products (one being R112), compared with two recovered in the same progeny in the more distal region, spanning the remaining 23% telomeric end of the arm (Ceoloni et al., 2005). For its consistent behavior in homologous and homoeologous contexts, the 5% 7el_1_L stretch included in R112 appears as a recombination hotspot, similar to several others frequently mapped to subterminal regions in wheat and related Triticeae chromosomes (e.g., Saintenac et al., 2011; Zhang et al., 2018).

### Efficacy of the *Fhb-7EL* QTL and Value of Novel Recombinant Types

In view of its exploitation in durum wheat breeding, verification of the full expression of the *Fhb-7EL* resistance QTL into the target species background was an essential step. All evaluation parameters, from assessment of FHB severity following controlled inoculations, to measurement of seed setting and development and, importantly, quantitation of DON content, provided consistent evidence of remarkably high reduction of all symptoms and effects of *F. graminearum* infection in genotypes carrying *Fhb-7EL* as compared with non-carrier lines. As confirmed by inclusion of the CS7E(7D) bread wheat substitution line in the infection assay (**Figure 3A**), the over 90% reduction of the FHB severity in inoculated spikes of novel 4x recombinant lines was of the same extent than that observed in the bread wheat background (**Figure 3A** and Ceoloni et al., 2017a), and even higher than that provided to both 6x and 4x wheat by the *Th. ponticum* 7el_2_ QTL (see also Introduction), averaging 80% (Forte et al., 2014). Transfer of the *Fhb1* major resistance QTL from Sumai 3 into *T. durum* cultivars led to a reduction of FHB severity from 6 to 36%, depending on the background (Prat et al., 2017). Moreover, undesirable effects on agronomic traits were reported when using Sumai 3 in breeding efforts (reviewed in Gilbert and Haber, 2013). On the other hand, particularly accurate and complex selection strategies are needed to effectively exploit multiple small-effect QTL (Tuberosa and Pozniak, 2014; Miedaner et al., 2017; Sari et al., 2018; Steiner et al., 2019).

By contrast, the completely dominant expression of a single major QTL, for whose selection a single PCR assay is sufficient, undoubtedly represents the ideal, breeder-friendly situation, and this is offered by the *Fhb-7EL* QTL. This locus has been shown to confer the same protection against FHB to different bread wheat lines, from the standard CS to the Italian elite cultivar Blasco (Ceoloni et al., 2017a), and to unrelated durum wheat genotypes, such as the Italian cv. Simeto (this work) and Langdon, an old North Dakota variety and laboratory line to which the complete chromosome 7E was recently added (Liu et al., 2017). Moreover, comparing the resistance demonstrated by the latter work with the results presented here confirms that the exceptional FHB resistance associated with *Th. elongatum* 7E chromosome is completely determined by the *Fhb-7EL* locus previously mapped to the distal end of 7EL (Ceoloni et al., 2017a).

Visual assessments of head blight were fully consistent with the prominently reduced accumulation of the DON toxin (**Table 6**), hence considerably reducing the health risk from FHB infection of the novel materials. Whether the *Fhb-7EL*-linked low DON content is due to its more efficient *in planta* conversion into the less active DON-3-glucoside derivative, which is identified as the main detoxification strategy in wheat and correlated to the *Fhb1* resistant response (e.g., Kluger et al., 2015; Lemmens et al., 2016; Mandalà et al., 2019 and references therein), or to alternative mechanisms (e.g., Miller et al., 2011), remains to be elucidated. Near-isogenic lines of durum wheat recombinants with and without *Fhb-7EL*, currently under development, will be ideal tools for comparative analyses aimed at elucidating the mechanism(s) of action underlying this unique resistance gene.

Among the intriguing characteristics of the *Fhb-7EL* QTL is its efficacy also toward another important Fusarium disease, i.e., crown rot (FCR), both in bread wheat (Ceoloni et al., 2017a) and in durum wheat (this work). Particularly prevalent in semi-arid regions amenable to the latter crop, FCR is increasingly showing an upsurge in incidence and severity in durum wheat, thereby causing even higher yield losses than in other susceptible cereals (GRDC Grains Research and Development Corporator, 2009; Fernandez and Conner, 2011; Scherm et al., 2013; Chekali et al., 2016). Preliminary evidence on durum wheat recombinant lines carrying the FHB resistance QTL from *Th. ponticum* 7el_2_ (Forte et al., 2014) similarly showed that QTL to confer resistance also to FCR (Ceoloni et al., unpublished results). Effectiveness toward both diseases, incited by different *Fusarium* species (*F. graminearum*, *F. culmorum* and *F. pseudograminearum*; see also Ceoloni et al., 2017a), is an exceptional attribute of the *Thinopyrum* spp. QTL, not paralleled by the situation in wheat germplasm, within which such a genetic and phenotypic coincidence finds no clear-cut example (Li et al., 2010). The largely comparable phenotype, combined with the corresponding location at the most distal end on the respective arms, 7el_2_L and 7EL (see Forte et al., 2014 and Ceoloni et al., 2017a), suggests the *Th. ponticum* and *Th. elongatum* Fusarium resistance QTL to be orthologous. Whereas high resolution maps of the respective chromosomal regions will be a necessary tool to verify this hypothesis, comparison of the gene content of the distal portions of *Th. ponticum* 7elL and *Th. elongatum* 7EL reveals additional similarities, including a *Psy1* gene, a likely candidate for the “yellow pigment” phenotype, common to 7el_1_L, 7el_2_L and 7EL (Forte et al., 2014, Ceoloni et al., 2017a; see also Introduction), and *Sd* (segregation distortion) genes spread along the arms, particularly in their proximal halves (Ceoloni et al., 2014b, Ceoloni et al., 2017b).

Regarding the effect of *Thinopyrum Psy1* alleles, this work has offered for the first time the possibility to assess the relative strength of *Psy1-7el* *_1_* *L* from *Th. ponticum* and *Psy1-7EL* from *Th. elongatum* once inserted into durum wheat. In contrast to what observed at the bread wheat level, where both contributed to a YPC increase, with the former providing a more conspicuous effect than the latter (Ceoloni et al., 2017a), only *Psy1-7el* *_1_* *L* was found to determine a significant increment of semolina YI in durum wheat. A likely explanation for what resulted in the different species contexts could be that the effect of the weaker *Psy1-7EL* allele can be detected when it replaces the non-contributing *Psy-D1a* allele, foremost widespread in bread wheat worldwide collections (Ravel et al., 2013), but not when it substitutes for alleles at the *Psy-A1* locus, as in the present durum wheat recombinant lines. Both in bread wheat (Ravel et al., 2013) and in durum wheat (Pozniak et al., 2007) major QTL for YPC have been mapped on 7AL and 7BL arms, which co-locate with *Psy1* alleles. The evidently stronger effect on semolina yellowness of *Psy1-7el* *_1_* *L* over the resident *Psy1-7AL* allele (see also Gennaro et al., 2007) confers to the R69-9 derivatives a particularly desirable attribute for transformation into pasta products.

The preliminary field trials showed no penalty on yield-related traits at the plant and spike levels associated with presence of either 7el_1_L+7EL segment. Instead, positive effects on spike fertility traits of both R74-10/R112 and R69-9/R112 recombinant lines (notably grain number per spike and per spikelet) were mostly evident in the 2^nd^ year trial (**Table 7B**). In both experimental seasons, the presence of the 7el_1_L leaf rust resistance gene *Lr19*, initially tracked by the STS*Lr19*_130_ closely linked marker, was validated in field grown plants. Its remarkable and durable efficacy is an additional, important asset in sustainable breeding.

While larger-scale and multi-location field trials are planned to better evaluate yield-related characteristics of all novel recombinant types, their highly valuable package of genes/QTL has already prompted marker-assisted crossing programs to incorporate the composite *Thinopyrum* segment into elite durum wheat varieties of different geographical origin. Further, the R5- or R112-type segments, involving a 7A chromosome, are being transferred into bread wheat as well, to evaluate their relative performance as compared with that of 7D recombinants previously engineered with the same 7EL portions but in a much longer 7el_1_L segment (Ceoloni et al., 2017a).

In conclusion, the chromosome engineering work described here marks a significant step forward in equipping durum wheat with highly desirable attributes, primarily the largely missing resistance to Fusarium diseases, which can sustainably enhance security and safety, as well as market and trade values of this important crop.

## Data Availability Statement

All datasets generated for this study are included in the article/supplementary material.

## Author Contributions

LK carried out most of the research, contributing to germplasm development, molecular and phenotypic selection and cytogenetic characterization; GM and ST performed the *Fusarium* spp. infection assays and analysed the data; RC and MF contributed to germplasm development and screening; RR and FR carried out the field test, the agronomic evaluations and statistical analyses; FG and SR performed the DON analysis. CC conceived the project, coordinated the research and prepared the manuscript. All authors read and approved the final manuscript.

## Funding

The research was partially supported by MIUR (Italian Ministry for education, University and Research) in the context of the initiative “Departments of excellence” (law 232/216), and by Lazio region, FILAS project “MIGLIORA”. 

## Conflict of Interest

The authors declare that the research was conducted in the absence of any commercial or financial relationships that could be construed as a potential conflict of interest.
